# Remarkable response to cardiac resynchronization therapy via left bundle branch pacing in patients with true left bundle branch block

**DOI:** 10.1002/clc.23462

**Published:** 2020-09-22

**Authors:** Jincun Guo, Linlin Li, Guosheng Xiao, Tao Ye, Xinyi Huang, Fanqi Meng, Qiang Li, Simei Chen, Binni Cai

**Affiliations:** ^1^ Division of Cardiology Xiamen Cardiovascular Hospital, Xiamen University Xiamen Fujian China; ^2^ Division of Echocardiography Xiamen Cardiovascular Hospital, Xiamen University Xiamen Fujian China; ^3^ Division of Cardiac Function Xiamen Cardiovascular Hospital, Xiamen University Xiamen Fujian China

**Keywords:** biventricular pacing, cardiac resynchronization therapy, heart failure, left bundle branch block, left bundle branch pacing, physiological pacing

## Abstract

**Background:**

Left bundle branch pacing (LBBP) has been suggested as an alternative means to deliver cardiac resynchronization therapy (CRT).

**Hypothesis:**

LBBP may deliver resynchronization therapy along with an advantage over traditional biventricular (BiV) pacing in clinical outcomes.

**Methods:**

Heart failure patients who presented LBBB morphology according to Strauss's criteria and received successful CRT procedure were enrolled in the present study. Propensity score matching was applied to match patients into LBBP‐CRT group and BiV‐CRT group. Then, the electrographic data, the echocardiographic data and New York heart association (NYHA) class were compared between the groups.

**Results:**

Twenty‐one patients with successful LBBP procedure and another 21 matched patients with successful BiV‐CRT procedure were finally enrolled in the study. The QRS duration (QRSd) was narrowed from 167.7 ± 14.9 ms to 111.7 ± 12.3 ms (*P* < .0001) in the LBBP‐CRT group and from 163.6 ± 13.8 ms to 130.1 ± 14.0 ms (*P* < .0001) in the BiV‐CRT group. A trend toward better left ventricular ejection fraction (LVEF) was recorded in the LBBP‐CRT group (50.9 ± 10.7% vs 44.4 ± 13.3%, *P* = .12) compared to that in the BiV‐CRT group at the 6‐month follow‐up. A trend toward better echocardiographic response was documented in patients receiving LBBP‐CRT procedure (90.5% vs 80.9%, *P* = .43) and more super CRT response was documented in the LBBP‐CRT group (80.9% vs 57.1%, *P* = .09) compared to that in the BiV‐CRT group.

**Conclusions:**

LBBP‐CRT can dramatically improve the electrical synchrony in heart failure patients with LBBB. Meanwhile, compared with the traditional BiV‐CRT, it has a tendency to significantly improve LVEF and enhance the NYHA cardiac function scores.

## 1 INTRODUCTION

Biventricular pacing (BiV) has been established as a credible adjuvant treatment for patients with heart failure and LBBB.^1^, ^2^, ^3^ However, approximately 30% of patients do not respond to cardiac resynchronization therapy (CRT) of standard BiV pacing. CRT nonresponse is multi‐factorial with suboptimal LV lead placement a dominant contributing factor and the electrical synchrony restored by BiV is actually a nonphysiological one, which is through variable fusion of wavefronts propagating from endocardium and epicardium.^4^ Left bundle branch pacing (LBBP) is a novel strategy for CRT which was first recommended by Huang et al in 2017.^5^ Later on, Zhang et al reported that LBBP was clinically feasible and effective in a small cohort of heart failure patients with LBBB.^6^ Recently, several nonrandomized studies suggested that LBBP‐CRT has some advantages in restoring electrical synchrony over BiV‐CRT leading to better CRT response. New criteria to define the presence of a “true” LBBB have been proposed by Strauss et al, which include a QS or rS morphology in V1 to V2, a duration ≥140 ms for men and ≥ 130 ms for women, along with mid‐QRS notching or slurring in ≥2 leads among I, aVL, V1, V2, V5, and V6.^7^ Studies had shown that patients with heart failure and LBBB met the Strauss's criteria had good response to BIV. More evidences are needed to confirm whether LBBP‐CRT is superior to BIV‐CRT in this special patient population. In this study, we evaluated the ability of LBBP to deliver resynchronization in patients with strictly defined LBBB and indications for CRT.

## 2 METHODS

### 2.1 Patient selection

This was a prospective, observational study. Heart failure patients presented LBBB morphology met Strauss's criteria, with left ventricular ejection fraction(LVEF) ≤35%, New York Heart Association (NYHA)functional class II to IV and successful CRT procedure in Xiamen Cardiovascular Hospital, Xiamen University from January, 2018 to December, 2019 were recruited in the present study. Since February 2018, LBBP was offered as an alternative choice for CRT in patients with typical LBBB in our center. The operator discussed the nonstandard but potentially more physiological pacing approach with the patients and obtained informed consent before the LBBP‐CRT procedure. All the patients that received successful LBBP‐CRT procedure were included. Patients who received successful BiV‐CRT during the study period were selected as control by using 1 to 1 propensity score matching to minimize bias based on gender, etiology and LV end‐diastolic diameter (LVEDD). Patients with previous pacemaker implanted, followed up irregularly or less than 6 months, and could not provide informed consent were excluded. The approval of the Ethics Committee of Xiamen Cardiovascular Hospital, Xiamen University was obtained prior to patient enrollment, and informed consent was obtained from all participants. The trial was conducted in accordance with the principles of the Declaration of Helsinki.

### 2.2 Procedural details

In the LBBP‐CRT group, the LBBP lead was implanted by a trans‐ventricular septal method in the basal ventricular septum as described elsewhere.^8^ Briefly, the Select Secure pacing lead(model 3830, Medtronic Inc., Minneapolis, Minnesota) was introduced through a fixed curve sheath (C315 HIS, Medtronic Inc.) anteroseptally toward the His region to localize His potentials. Temporary His bundle pacing(HBP) was attempted to determine whether LBBB could be corrected. Then the lead was further advanced toward the cardiac apex by 1.0 to 2.0 cm and perpendicularly screwed in. The lead was finally fixed when the paced QRS morphology showed a “QR/Qr” pattern in V1, the stimulus to LV activation time (stim‐LVAT) was the shortest and consistent during high and low outputs in V5 or V6, and the capture threshold <1.5 V@0.4 ms^9^. If the LBBP procedure was unsuccessful, traditional BiV‐CRT was selected as an alternative pacing modality.

In the BiV‐CRT group, a LV lead was positioned with standard technique in the lateral or post‐lateral LV vein if possible.^10^ RV defibrillator lead or pacing lead was implanted in the right ventricular apex and atrial lead was implanted in the right atrial appendage (RA) in patients with sinus rhythm.

### 2.3 Device connection and programming

**FIGURE 1:**
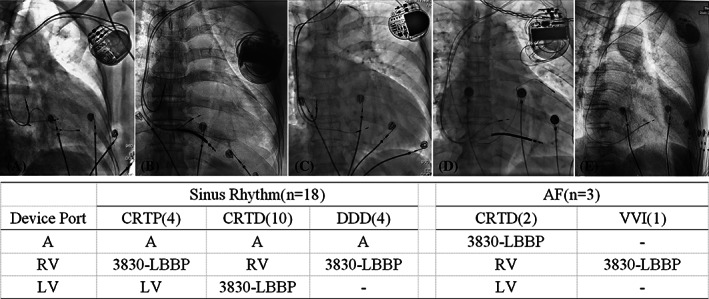
The lead to device connection configurations A: right atrial port. AF, atrial fibrillation; LBBP, left bundle branch pacing; LV, left ventricular port; RV, right ventricular port

In the LBBP‐CRT group, CRT‐pacemakers (CRT‐P) and CRT‐defibrillators (CRT‐D) were the first choice for implantation, while DDD or VVI implantation was selected as an alternative in patients with poor economic conditions on the late period of the study. The lead to device connection configurations are showed in Figure 1. All LBBP leads were programmed at tip unipolar pacing with output of 3.0 V@0.4 ms. In patients with sinus rhythm, the PAV/SAV was adjusted to synchronize LBBP with intrinsic right bundle branch conduction in the LBBP‐CRT group，while the PAV/SAV was programmed to 130/100 ms and V‐V delay was programmed to LV‐RV 20 ms in the BiV‐CRT group.

### 2.4 Data collections and follow up

Baseline characteristics and medical history of participants were collected at enrollment. Twelve‐lead surface ECG and the intracardiac electrogram (IEGM) were recorded by the GE CardioLab Electrophysiology recording system (GE Healthcare Inc., Marlborough, Massachusetts) at 100 mm/sec. The intrinsic QRSd, the paced QRSd (pQRSd), the stim‐LVAT and the QT interval were measured in sequence. The QRSd was measured from the first onset of the complex to the latest offset in all leads. Stim‐LVAT was measured from the stimulus to the peak of R‐wave in lead V5 or V6. The QT interval was measured from the QRS onset at the earliest deflection in any lead to the end of the T wave in any lead, and the QTc was calculated using the Bazett formula as the duration of the QT interval adjusted for the patient's heart rate.^11^ The echocardiographic parameters were documented including LVEF, LVEDD and LV end systolic dimension (LVESD) The LVEF was calculated with the modified Simpson's method (Three measures from each projection were taken, and the average value was recorded). All echocardiographic examinations were performed by the same experienced echocardiographer and analyzed by two independent experienced echocardiographers blinded to the study.

All patients were followed up at 3 months, 6 months and 1 year after implantation. The data of patients' survival status, NYHA functional class, and echocardiographic data were routinely documented and collected. Lead parameters including pacing threshold, R‐wave amplitude and impedance were recorded at implantation and each follow‐up visit. Possible complications such as infections, pericardial effusion, chronic capture threshold elevation, lead dislodgment and lead deficiencies were routinely tracked.

### 2.5 Definition of CRT response

Echocardiographic response was defined as an LVEF improvement of at least 5% at the 6‐month follow‐up visit compared to that at baseline. CRT response was defined as an improvement of the patient's symptomatic status, such as decreasing NYHA functional class for at least one grade at the last follow‐up compared to the basal value. CRT super‐response was defined as a significant improvement in heart function, with the NYHA functional class decreasing to grade I or II, along with greater improvement in LVEF for at least 15% or a final LVEF>45%, and a decrease in LVESD>15%.^12^

### 2.6 Statistical analysis

Descriptive statistics were used to summarize the clinical and echocardiographic outcomes. Continuous variables were expressed as the mean ± SD, and categorical variables were presented as the numbers and percentages in each group. In propensity matched analysis, the variables in the logistic regression model for calculating propensity score (PS) included gender, etiology and LVEDD. Matching was performed using the logit‐transformed PS. An optimal matching algorithm with a caliper width of 0.2 standard deviations of the logit of the PS was used. Comparison of continuous variables was tested by Student's *t* test or Mann‐Whitney *U*‐test according to the data distribution. Comparisons of categorical variables were compared by Chi‐square analysis. All statistical tests were two tailed and a value of *P* < .05 was considered statistically significant. All statistical analyses were performed using SPSS Statistics version 22.0 (Chicago, IL, USA).

## 3 RESULTS

### 3.1 Study population

A total of 24 patients underwent LBBP‐CRT attempt during this study period, among them 21 were successful in the LBBP procedure resulting in a success rate of 87.5% (supplement data). Three patients failed in LBBP‐CRT procedure due to difficulties in screwing the 3830 lead into the deep ventricular septum and subsequent BiV‐CRT was successfully performed as an alternative. Twenty‐one matched patients with successful BiV‐CRT were recruited as control. The mean age was 65.6 ± 8.7 years (range 45 to 82 years), 18 patients (42.9%) were male and 38 patients (90.5%) were diagnosed with nonischemia cardiomyopathy (NICM) in this cohort. Detailed baseline characteristics of the recruited patients in both groups were described in Table 1. No significant differences were noted between LBBP‐CRT group and BiV‐CRT group based on baseline demographics, echocardiographic measurements and medication.

**TABLE 1:** Baseline characteristics of the recruited patients

	LBBP(N = 21)	BIV(N = 21)	*P* value
Age, y mean ± SD	66.1 ± 9.7	65.1 ± 7.5	.59
Male, n(%)	9(42.9%)	9(42.9%)	1.0
NICM n (%)	19(90.5%)	19(90.5%)	1.0
ICM，n (%)	2 (9.5%)	2 (9.5%)	1.0
HT	9(42.9%)	7(33.3%)	.52
DM	8(38.1%)	1(4.8%)	.08
CKD	3(14.3%)	1(4.8%)	.29
AF, n (%)	3(14.3%)	1(4.8%)	.29
Intrinsic QRSd(ms)	167.7 ± 14.9	163.6 ± 13.8	.36
LVEDD(mm)	64.9 ± 7.2	66.7 ± 5.4	.36
LVEF(%)	30.0 ± 5.0	29.8 ± 4.1	.77
MR:			.07
Mild， n(%)	8(38.1%)	10(47.6%)	
Moderate， n (%)	7(33.3%)	6(28.6%)	
Severe， n (%)	4(19.1%)	3(14.3%)	
NYHA class			.64
II，n (%)	4(19.1%)	4(19.1%)	
III，n (%)	10(47.6%)	12(57.1%)	
IV，n (%)	7(33.3%)	5(23.8%)	
Medicine			
ACEI/ARB/ARNI	19(90.5%)	19(90.5%)	1
Beta‐blockers	20(95.2%)	21(100%)	.31
Aldosterone antagonist	18(85.7%)	21(100%)	.07
Diuretics	18/(85.7%)	21(100%)	.07
Digoxin	10(47.6%)	13(61.9%)	.35
Amiodarone	1(4.8%)	5(23.8%)	.07
Ivabradine	3(14.3%)	6(28.6%)	.26

Abbreviations: AF, atrial fibrillation; BiV, biventricular pacing; CKD, chronic kidney disease; DM, diabetes; HT, hypertension; ICM, ischemic cardiomyopathy; LBBP, left bundle branch pacing; LVEDD, left ventricular end diastolic diameter; LVEF, left ventricular ejection fraction; MR, mitral valve regurgitation; NICM, nonischemic cardiomyopathy; NYHA class, the New York heart association functional class.

### 3.2 Procedural outcomes

Among the 24 patients underwent LBBP‐CRT attempt, His potential was documented in 17 patients (70.8%)and LBBB was corrected in 15 out of this 17 patients (88.2%) undergoing temporary HBP. LBBP was successful in 21 patients with LBBB corrected under low output, and the mean LBBP capture threshold was 0.48 ± 0.22 V@0.4 ms. The mean pQRSD was 116.7 ± 9.7 ms and the mean stim‐LVAT were 81.2 ± 13.2 ms under LBBP with tip unipolar pacing. Twelve patients received CRT‐D and four patients received CRT‐P implantation. DDD were implanted in four patients and VVI was implanted in one patient who was complicated with atrial fibrillation, in lieu of tri‐chamber pacing due to poor economic conditions (supplement data), LBBP synchronized with intrinsic right bundle branch conduction and the shortest QRSd was achieved by adjusting AV delay in 18 patients with sinus rhythm, except for three patients with persistent atrial fibrillation. In the BiV‐CRT group，the mean LV capture threshold was 1.12 ± 0.46 V@0.4 ms. Twelve patients received CRT‐D implantation and nine patients received CRT‐P in this group. LV lead were positioned in the lateral LV vein in 14 patients, in the post‐lateral LV vein in one patients and anterior‐lateral LV vein in six patients. The X‐ray exposure duration were significantly shorter in the LBBP‐CRT group than that in the BiV‐CRT group (17.9 ± 7.1 minutes vs 37.8 ± 14.2, *P* < .001).

### 3.3 Electrographic data

**FIGURE 2:**
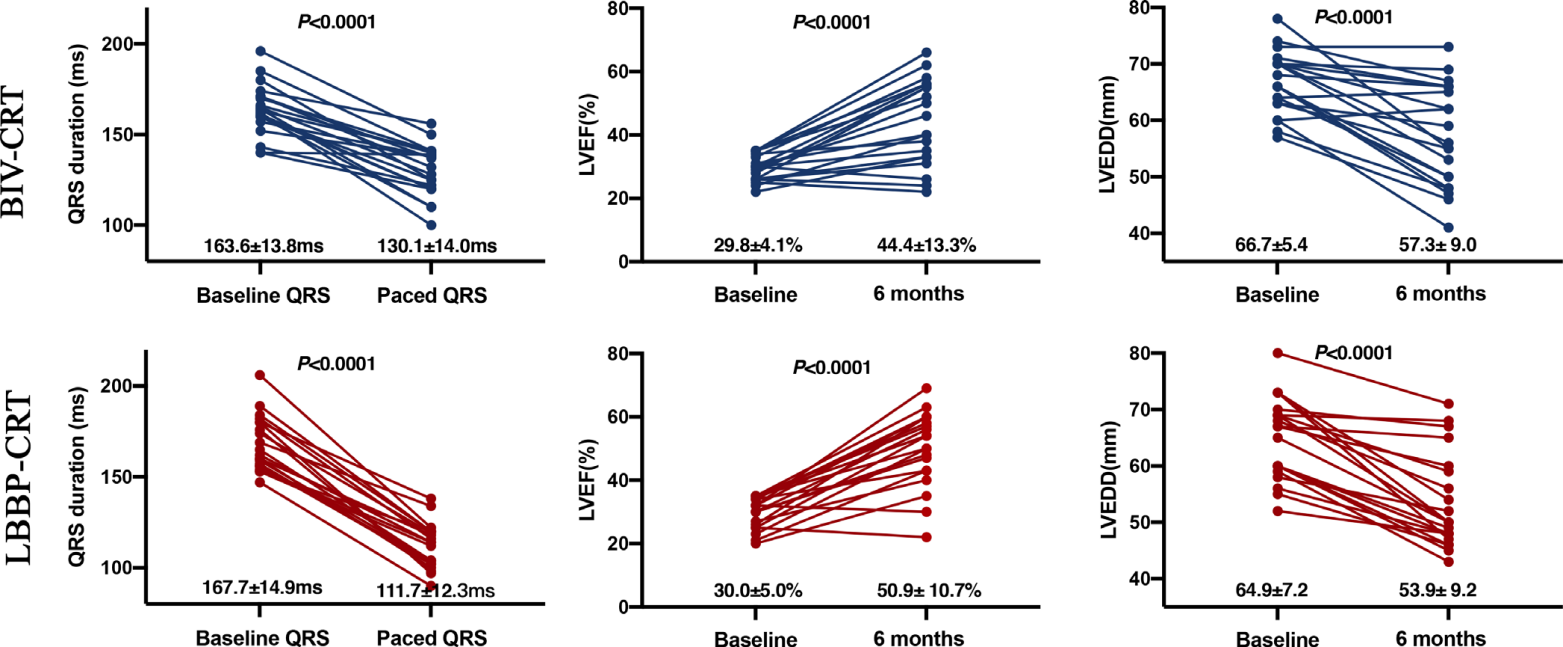
Change of QRSD, LVEF and LVEDD in LBBP‐CRT and BiV‐CRT. Pre and post QRS, LVEF, and LVEDD by individual patient in patients receiving LBB‐CRT vs BiV‐CRT were shown. BiV, biventricular pacing; CRT: cardiac resynchronization therapy; LBBP, left bundle branch pacing; LVEF: left ventricular ejection fraction; LVEDD, left ventricular end diastolic dimension

The detailed electrographic data were shown in Table 2. There were no significant differences in QRSd, and QTc at baseline between the LBBP‐CRT group and the BiV‐CRT group. The paced QRSd and QT interval were narrowed significantly compared to the baseline value in both CRT groups. The QRSd was narrowed from 167.7 ± 14.9 ms to 111.7 ± 12.3 ms (*P* < 0.0001) after optimizing AV delay in the LBBP‐CRT group, while the QRS duration was narrowed from 163.6 ± 13.8 ms to 130.1 ± 14.0 ms (*P* < .0001) in the BiV‐CRT group. The reduction in QRSd was more remarkable in the LBBP‐CRT group compared to that in the BiV‐CRT group (Figure 2).

**TABLE 2:** Comparison of electrographic data and outcomes n different groups

	LBBP‐CRT	BIV‐CRT	*P* value
VP%	99.1 ± 1.9%	99.3 ± 0.9%	.67
Electrocardiographic data			
QRSD			
intrinsic QRSD (ms)	167.7 ± 14.9	163.6 ± 13.8	.36
Paced QRSD (ms)	111.7 ± 12.3^a^	130.1 ± 14.0^a^	<.0001
⊿QRSD (ms)	56.0 ± 14.7	32.3 ± 14.6	<.0001
⊿QRSD / intrinsic QRSD(%)	33.4 ± 7.2	19.6 ± 8.5	<.0001
QTC			
intrinsic QTC (ms)	515.7 ± 32.3	512.2 ± 41.1	.8
Paced QTC (ms)	450.9 ± 31.5^a^	474.3 ± 33.5^a^	.06
Echocardiographic data			
LVEDD(mm)			
Baseline	64.9 ± 7.2	66.7 ± 5.4	.36
6 months	53.9 ± 9.2^b^	57.3 ± 9.0^b^	.15
LVESD(mm)			
Baseline	53.8 ± 9.5	55.0 ± 7.4	.06
6 months	39.5 ± 10.7^b^	44.5 ± 11.1^b^	.15
LVEF(%)			
Baseline	30.0 ± 5.0	29.8 ± 4.1	.77
6 months	50.9 ± 10.7^b^	44.4 ± 13.3^b^	.12
⊿LVEF	20.5 ± 9.6	15.4 ± 11.2	.15
Clinic response			
NYHA class			
Baseline	3.0 ± 0.7	3.0 ± 0.7	.64
6 months	1.3 ± 0.9^b^	1.5 ± 0.7^b^	.06
Improvement≥1 class	21 (100%)	19 (90.4%)	.15
Improvement≥2 class or improvement to I class	18 (85.7%)	13 (61.9%)	.08
CRT response			
Response rate	19 (90.5%)	17 (80.9%)	.43
Super response rate	17 (80.9%)	12 (57.1%)	.09

^a^ *P* < .01, compared with intrinsic conduction in the same group.

^b^ *P* < .0001, comparison between baseline and 6 months follow‐up within the same group.

Abbreviations: BiV, biventricular pacing; CRT, cardiac resynchronization therapy; LBBP: left bundle branch pacing; LVEDD, left ventricular end diastolic diameter; LVSED, left ventricular end systolic diameter; LVEF, left ventricular ejection fraction;⊿LVEF, change in absolute LVEF between baseline and 6 months; QRSD, QRS duration；⊿QRSD, reduction in QRS duration before and after pacing in the same patient; NYHA class, the New York heart association functional class.

### 3.4 Clinical outcomes

The mean follow‐up period was 14.3 ± 7.2 months (ranging from 6 to 27 months). Significant improvements were recorded with respect to baseline measurements for NYHA Class and echocardiographic data at 6 month follow‐up in both groups (Figure 2). All the participants completed the 6 months follow‐up with LVEF improved from 30.0 ± 5.0% to 50.9 ± 10.7% (*P* < .0001), and NYHA Class from 3.0 ± 0.7 to 1.3 ± 0.9 (*P* < .001) in the LBBP‐CRT group. Detailed echocardiographic data were shown in Table 2. Although no statistically significant difference, a trend toward better LVEF (50.9 ± 10.7% vs 44.4 ± 13.3%, *P* = .12) along with better echocardiographic response (90.5% vs 80.9%, *P* = .43) and more super CRT‐response (80.9% vs 57.1%, *P* = .09) was documented in the LBBP‐CRT group compared to that in BiV‐CRT group. Event rates were assessed during the follow‐up. No heart failure hospitalization, VT/VF or all‐cause deaths were documented in both groups.

### 3.5 Lead parameters

**FIGURE 3:**
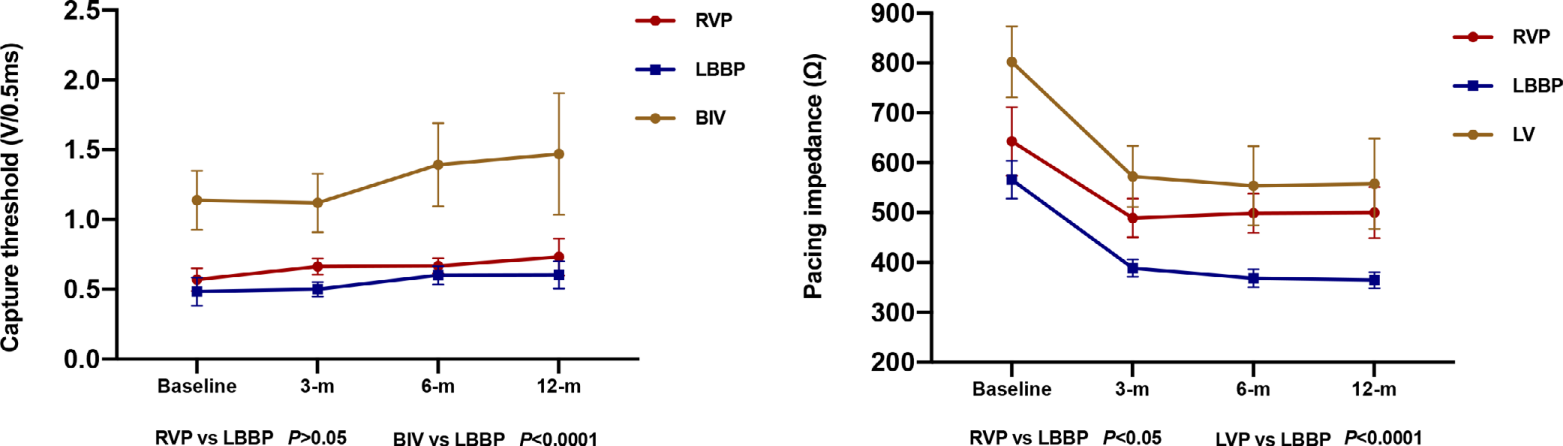
Comparison of pacing parameters in LBBP, RVP, and LVP during follow‐up. BiV, left ventricular pacing lead; RVP, right ventricular pacing lead; LBBP, left bundle branch pacing lead; LVP, left ventricular pacing

The capture threshold for LBBP lead was significantly lower than that of LV lead(0.48 ± 0.22 V@0.4 ms vs 1.12 ± 0.46 V@0.4 ms，*P* < 0.0001)while similar to that of right ventricular lead (0.48 ± 0.22 V@0.4 ms vs 0.57 ± 0.17 V@0.4 ms, *P* = .17)during the procedure, and the difference in threshold remained throughout the whole observation period (Figure 3). The capture threshold for LBBP lead remained stable in all patients during follow‐up, while chronic capture threshold elevation was observed at 2.5 V@0.4 ms in two patients in the BiV‐CRT group. Lead impedance declined significantly after the acute phase and remained stable throughout the whole observation period in all patients.

### 3.6 Complications

Procedure‐related RBB injuries, resulting in transient three degree AVB occurred in four patients in the LBBP‐CRT group and in one patient in the BiV‐CRT group during implantation, which disappeared within 24 hours in all cases. No complications such as device or lead infections, lead revision, chronic capture threshold elevation and aorta or coronary artery injury were documented in the LBBP‐CRT group. Phrenic nerve stimulation (PNS) was documented in two patients due to cardiac remodeling in the BiV‐CRT group and was ameliorated by downgrading the outputs.

## 4 DISCUSSION

In the present study, we compared the clinical outcomes of LBBP‐CRT and BiV‐CRT in patients with strictly defined LBBB and indications for CRT. We demonstrate that: (a). LBBP‐CRT was superior to BiV‐CRT in electrical resynchronization (b). A trend toward greater increment in LVEF and better improvement in NYHA class could be achieved under LBBP‐CRT compared to BiV‐CRT (c). LBBP‐CRT procedure is safe and feasible accompany with low LBBB correction threshold and stable intermediate‐term lead parameters. We believe that the finding derived from the present study could provide justification for planning powered randomized controlled trials to delineate the clinical benefits with this new pacing modality.

Conduction system pacing has emerged as a feasible pacing strategy for CRT in heart failure patients with LBBB.^13^ Various studies have demonstrated that HBP can deliver better electrical synchrony compared to BiV‐CRT, resulting in improvement of LVEF, life quality and NYHA functional class.^14^, ^15^, ^16^, ^17^ However, HBP is technically challenging due to its anatomical location and high capture threshold..^18^ In the largest prospective study with HBP‐CRT conducted by Huang et al., LBBB correction was achieved in 96.6% participants with strictly defined LBBB. However, permanent HBP could achieve in only 76% patients as a result of high LBBB correction thresholds or difficulties in lead fixation.^19^ Due to the distal location of the LBB lead in the left His‐Purkinje system, a stable and low pacing capture threshold under LBBP is feasible, especially in situations where the pacing circumvents a proximal site of block.^20^ In our cohort, successful permanent LBBP was achieved in 88% patients with low and stable capture threshold. The capture threshold was much lower and remained stable during follow‐up compared to that under HBP in previous studies.^21^

The LBB fans out directly from the branching point of His bundle to form a network structure beneath the endocardium of the left side of the interventricular septum, which promises pacing the LBB feasibly and rapidly through an interventricular septal method with significant QRS duration shortening.^22^ In the present study, better electrical resynchronization was achieved by LBBP‐CRT than BiV‐CRT. Meanwhile, better repolarization homogeneity, which was confirmed by shorter QTc, was restored by LBBP‐CRT. Several studies have revealed that prolonged paced QTc interval could serve as a predictor of risk for ventricular arrhythmias during CRT with an optimal cut‐off point of 485 ms.^23^, ^24^ The question of whether better depolarization or repolarization homogeneity maintained by LBBP‐CRT can translate to lower rate of iatrogenic arrhythmia should be answered with future studies.

It has been convinced that the degree of shortening of QRSd, is proportional to the improvement in cardiac function.^25^ In the present study, a trend toward better echocardiographic responses was documented in the LBBP‐CRT group with the CRT response rate in 90% and super response rate in 80%. The advantage of clinical benefits was not statistically significant in the present study due to relatively small sample size and short observation period. Powered randomized trials with larger sample size are needed to verify the results in the future.

Bypassing the conduction block at the proximal left conduction bundle and rapid left His‐Purkinje system recruitment is the cornerstone underlining LBBP resynchronization therapy in restoring electrical synchrony. It's important to recruit patients with “true” LBBB to ensure LBBB correction and evaluate clinical outcome during LBBP‐CRT. Evidences had confirmed that Strauss criteria was a better predictor to“true”LBBB than the traditional criteria accordance with ACC/AHA/HRS definition and participants met the Strauss's criteria had better response to BIV‐CRT.^7^, ^26^ Recently, Li et al. reported that LBBP‐CRT was successful in 81.1% of the heart failure patients with LBBB, and the response rate and super response rate were 88.9% and 44.4%, respectively, which were greater than that of BiV‐CRT(66.7% and 16.7%).^27^ However, the study wasn't randomizedly designed and the morphological characteristics of LBBB were not strictly defined in their study there may be more patients with“true”LBBB in the LBBP group and this may lead to an overestimation of the efficacy of LBBP‐CRT compared to BIV‐CRT. Different from their study, Wu et al strictly recruited patients with LBBB according to Strauss's definition and compared the echocardiographic response delivered by LBBP‐CRT, HBP‐CRT or BiV‐CRT.^28^ In their study, the rate of super response to BIV‐CRT was 53% and LBBP‐CRT was 77%. Another multicenter prospective cohort study conducted by Huang et al also confirms the high rate of super response under LBBP‐CRT in patients with strictly defined LBBB, which was consistent with the present study.^29^

The present study confirmed a better electrical synchrony and remarkable clinical outcomes of LBBP‐CRT in heart failure patients complicated with Strauss defined LBBB. Beyond that, LBBP also showed an advantage in low and stable capture threshold over traditional LV pacing during follow‐up. Given the advantage of relatively simple procedure, physiological pacing modality, long‐term stability of lead parameters, lower power consumption and potential benefit of battery longevity, this resynchronization modality is worthy of promotion in daily practice.

## 5 LIMITATION

The present study was a nonrandomized study conducted with a relatively small sample size in a single center. Propensity score matching was employed in the current study to correct for selection bias. In the present study, only a trend toward better echocardiographic response was documented in patients receiving LBBP‐CRT compared with BiV‐CRT. This may due to a relatively small sample size. Powered randomized trials with larger sample size are still needed to verify the results from the present study. The majority of patients recruited had nonischemic cardiomyopathy and thus the study cohort may not broadly represent the heart failure population in daily practice. Though better repolarization homogeneity was shown under LBBP‐CRT, differences in iatrogenic arrhythmia could not be demonstrated, which might be due to the relatively small sample size and a short observation period.

## 6 CONCLUSION

Compared with BiV‐CRT, LBBP‐CRT was remarkable in restoring electrical synchrony in heart failure patients complicated with strictly defined LBBB. A trend toward greater increment in LVEF and NYHA class was documented in this cohort underwent LBBP‐CRT. LBBP‐CRT procedure is safe and feasible, with low LBBB correction threshold and stable medium‐term lead parameters during follow‐up.

## CONFLICT OF INTEREST

The authors declared that they have no conflict of interest.

## AUTHOR CONTRIBUTIONS

Binni Cai conceived and designed the experiment. Jincun Guo and Linlin Li recruited the subjects and collected the clinical data. Tao Ye, Xinyi Huang, Qiang Li and Simei Chen conducted the laboratory testing. Guosheng Xiao and Fanqi Meng helped analyze the data. Jincun Guo and Linlin Li wrote the manuscript. All authors read and approved the final manuscript.

## ETHICAL STATEMENT

The study was approved by the Ethics Committee of Xiamen Cardiovascular Hospital, Xiamen University and was performed in line with the principles of the Declaration of Helsinki.

## INFORMED CONSENT

Informed consent was obtained from all participants.

## Supporting information


**Table S1** Baseline characteristics of the recruited patients

**Figure S1** LBBP‐CRT in a patient with NICM and strictly defined LBBB
A 65‐year‐old male heart failure patient presented a strict LBBB morphology. (a) the QRSd was 165 ms at baseline. LBBB was corrected under temporary HBP with high output (10 V@0.4 ms) and the stim‐LVAT was narrowed to 90 ms. LBBB could be corrected under high and low output with consistency in stim‐LVAT of 87 ms. Selective‐LBBP morphology presented with an isoelectric interval between the pacing spike and the QRS onset under low output, the HBP lead (solid arrow) and LBBP lead (hollow arrow) was shown under fluoroscopy. (b) The ECG morphology met the criteria for strict LBBB proposed by Strauss et al. (c) QRS duration narrowed from 165 ms to 116 ms under LBBP with SAVD 100 ms and the CTR significantly decreased from 0.66 to 0.48 with LVEF improved from 32% to 69% at 3‐month follow‐up.
NICM: non‐ischemic cardiomyopathy; LBBP: left bundle branch pacing; CTR: cardiothoracic ratio; LVEF: left ventricular ejection fraction; LVEDD: left ventricular end diastolic dimension, SAVD: sensed AV delay.

**Figure S2** LBBP‐CRT procedure in a patient with VVI implantation
A 48‐year‐old male heart failure patient complicated with atrial fibrillation received LBBP‐CRT procedure with VVI implantation due to poor economic condition. (a) a strict LBBB morphology presented with QRSd of 162 ms. LBBB could be corrected by HBP under high output (5 V@0.4 ms) and stim‐LVAT was 84 ms. LBBB could be corrected by LBBP under high output (5 V@0.4 ms) and low output (0.4 V@0.4 ms) with consistency in stim‐LVAT of 72 ms. (b) The ECG showed atrial fibrillation and strict LBBB morphology (c) QRS duration was narrowed from 162 ms to 114 ms under LBBP and the CTR decreased from 0.59 to 0.49 with LVEF improving from 35% to 57% at 6‐month follow‐up.

**Figure S3** Septal remodeling in a patient after LBBP‐CRT procedure
A patient with NICM and LBBB demonstrated super response to LBBP. The QRSd was narrowed from 198 ms to 120 ms and the CTR decreased from 0.65 to 0.50.The phenomenon of “lead protruding” toward the LV cavity disappeared after remodeling of the ventricular septum during follow‐ups. The patient's IVSd increased from 7.8 mm to 10.2 mm. A: pre‐operation; B: post‐operation.
LBBP: left bundle branch pacing; CTR: cardiothoracic ratio; LBBB: left bundle branch block; IVSd: interventricular septal diameter; NICM: non‐ischemic cardiomyopathy. QRSD: QRS duration.

## Data Availability

The datasets generated and/or analyzed during the current study are available from the corresponding author, Binni Cai, upon reasonable request.
