# Supplementary material for: Remarkable response to cardiac resynchronization therapy via left bundle branch pacing in patients with true left bundle branch block
Source: Clin Cardiol. 2020 Sep 22;43(12):1460–8. doi: 10.1002/clc.23462 (PMC7724211; doi:10.1002/clc.23462)
Supplement: Supplementary file 1 — Table S1 Baseline characteristics of the recruited patients [file CLC-43-1460-s001.docx]

| **Case** | **Baseline characteristics** | | | | | | | |  | **LBBP-Procedure** | | | |  | **Pacing QRSD(ms)** | |  | **Follow-up** | |
| --- | --- | --- | --- | --- | --- | --- | --- | --- | --- | --- | --- | --- | --- | --- | --- | --- | --- | --- | --- |
|  | **Sex** | **Age** | **Cause** | **Rhythm** | **Intrinsic QRSD (ms)** | **NYHA Class** | **LVEDD (mm)** | **LVEF (%)** |  | **Device** | **His potential recorded** | **LBBB corrected by HBP** | **LBBP Threshold V/0.4ms** |  | **LBBP (unipolar)** | **post AV Opti** |  | **Period (m)** | **VP%** |
| **1** | **F** | **77** | **DCM** | **SR** | **147** | **3** | **52** | **25** |  | **DDD** | **Y** | **Y** | **0.5** |  | **107** | **90** |  | **24** | **98.9** |
| **2** | **M** | **55** | **CAD** | **AF** | **169** | **4** | **67** | **27** |  | **CRTD** | **N** | **N** | **0.8** |  | **134** | **-** |  | **25** | **95.7** |
| **3** | **F** | **60** | **DCM** | **SR** | **206** | **4** | **80** | **22** |  | **CRTD** | **Y** | **Y** | **0.3** |  | **126** | **122** |  | **24** | **100** |
| **4** | **F** | **55** | **DCM** | **SR** | **157** | **3** | **56** | **26** |  | **CRT** | **Y** | **Y** | **0.9** |  | **100** | **87** |  | **24** | **100** |
| **5** | **F** | **82** | **DCM** | **SR** | **165** | **3** | **58** | **35** |  | **DDD** | **Y** | **N** | **0.6** |  | **107** | **104** |  | **24** | **100** |
| **6** | **M** | **82** | **DCM** | **SR** | **153** | **4** | **73** | **32** |  | **DDD** | **N** | **N** | **0.3** |  | **122** | **120** |  | **18** | **100** |
| **7#** | **M** | **50** | **DCM** | **SR** | **152** | **3** | **71** | **21** |  | **CRTD** | **Y** | **N** | **-** |  | **--** | **-** |  | **12** | **100** |
| **8#** | **F** | **45** | **DCM** | **SR** | **171** | **3** | **80** | **21** |  | **CRTD** | **Y** | **Y** | **-** |  | **--** | **-** |  | **14** | **99.9** |
| **9** | **F** | **71** | **DCM** | **AF** | **180** | **4** | **69** | **32** |  | **CRTD** | **Y** | **Y** | **0.4** |  | **134** | **-** |  | **16** | **97.9** |
| **10** | **M** | **66** | **DCM** | **SR** | **182** | **4** | **60** | **32** |  | **CRTD** | **Y** | **Y** | **0.3** |  | **120** | **116** |  | **12** | **99.9** |
| **11** | **M** | **52** | **DCM** | **SR** | **176** | **3** | **67** | **35** |  | **CRTD** | **Y** | **Y** | **0.6** |  | **100** | **98** |  | **12** | **98.2** |
| **12** | **F** | **55** | **DCM** | **SR** | **189** | **2** | **69** | **23** |  | **CRTD** | **Y** | **Y** | **0.6** |  | **125** | **120** |  | **12** | **99.6** |
| **13** | **F** | **61** | **DCM** | **SR** | **160** | **3** | **59** | **32** |  | **CRTD** | **N** | **Y** | **0.3** |  | **115** | **100** |  | **9** | **100** |
| **14** | **F** | **74** | **DCM** | **SR** | **153** | **4** | **60** | **30** |  | **CRTD** | **N** | **N** | **0.4** |  | **112** | **110** |  | **9** | **100** |
| **15** | **M** | **62** | **DCM** | **SR** | **154** | **3** | **65** | **35** |  | **CRTD** | **Y** | **Y** | **0.3** |  | **122** | **114** |  | **9** | **100** |
| **16** | **F** | **66** | **DCM** | **SR** | **156** | **2** | **55** | **35** |  | **CRT** | **Y** | **Y** | **0.5** |  | **107** | **102** |  | **9** | **99.6** |
| **17#** | **M** | **79** | **CAD** | **SR** | **182** | **3** | **63** | **35** |  | **CRTD** | **Y** | **Y** | **-** |  | **-** | **-** |  | **6** | **100** |
| **18** | **F** | **62** | **DCM** | **SR** | **174** | **4** | **70** | **24** |  | **DDD** | **N** | **N** | **0.4** |  | **114** | **119** |  | **6** | **99.7** |
| **19** | **M** | **82** | **CAD** | **SR** | **160** | **3** | **68** | **34** |  | **CRTD** | **N** | **N** | **0.8** |  | **120** | **118** |  | **6** | **99.9** |
| **20** | **M** | **75** | **DCM** | **AF** | **162** | **3** | **60** | **35** |  | **VVI** | **Y** | **Y** | **0.2** |  | **114** | **-** |  | **6** | **92.4** |
| **21** | **F** | **69** | **DCM** | **SR** | **180** | **3** | **73** | **30** |  | **CRTD** | **Y** | **Y** | **0.5** |  | **112** | **97** |  | **6** | **100** |
| **22** | **M** | **66** | **DCM** | **SR** | **159** | **2** | **60** | **34** |  | **CRTD** | **N** | **N** | **0.9** |  | **114** | **104** |  | **6** | **98.6** |
| **23** | **M** | **55** | **DCM** | **SR** | **156** | **2** | **69** | **35** |  | **CRT** | **Y** | **Y** | **0.2** |  | **120** | **103** |  | **6** | **99.9** |
| **24** | **F** | **61** | **DCM** | **SR** | **184** | **3** | **73** | **21** |  | **CRT** | **Y** | **Y** | **0.4** |  | **127** | **120** |  | **6** | **100** |

Table 1 Information list of all participants attempted to Left bundle branch pacing

# LBBP failed in Case7 ,Case8 & Case 17 and Biventricular pacing was performed as an alternative

NYHA class: the New York Heart Association functional class. LVEDD: left ventricular end diastolic diameter ;LVEF: left ventricular ejection fraction; LBBB: left bundle branch block, LBBP: left bundle branch pacing; AV Opti: AV Optimalization ; VP: ventricular pacing; Y yes , N no,
