# Supplementary figures and images for: Remarkable response to cardiac resynchronization therapy via left bundle branch pacing in patients with true left bundle branch block
Source: Clin Cardiol. 2020 Sep 22;43(12):1460–8. doi: 10.1002/clc.23462 (PMC7724211; doi:10.1002/clc.23462)

A

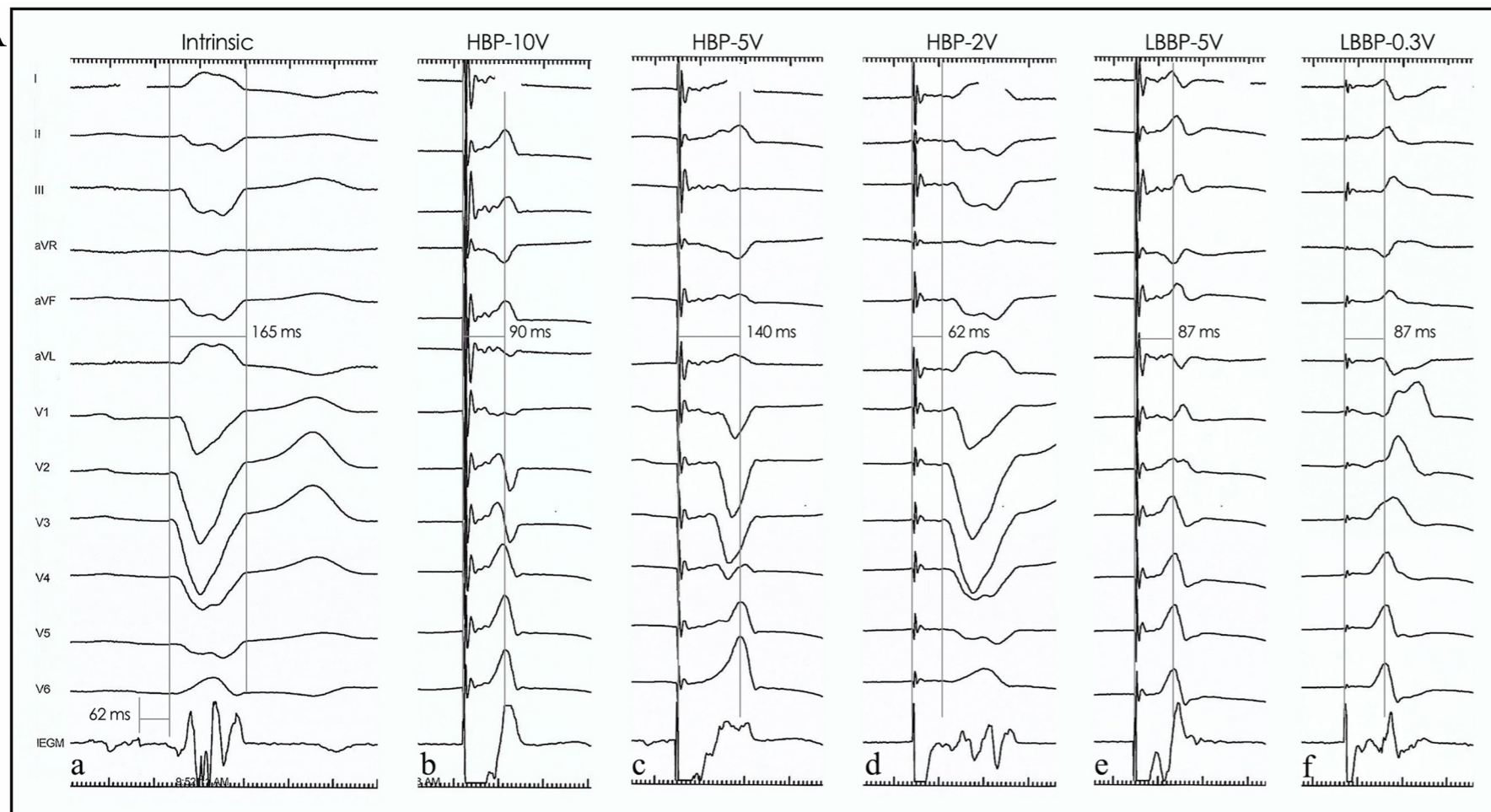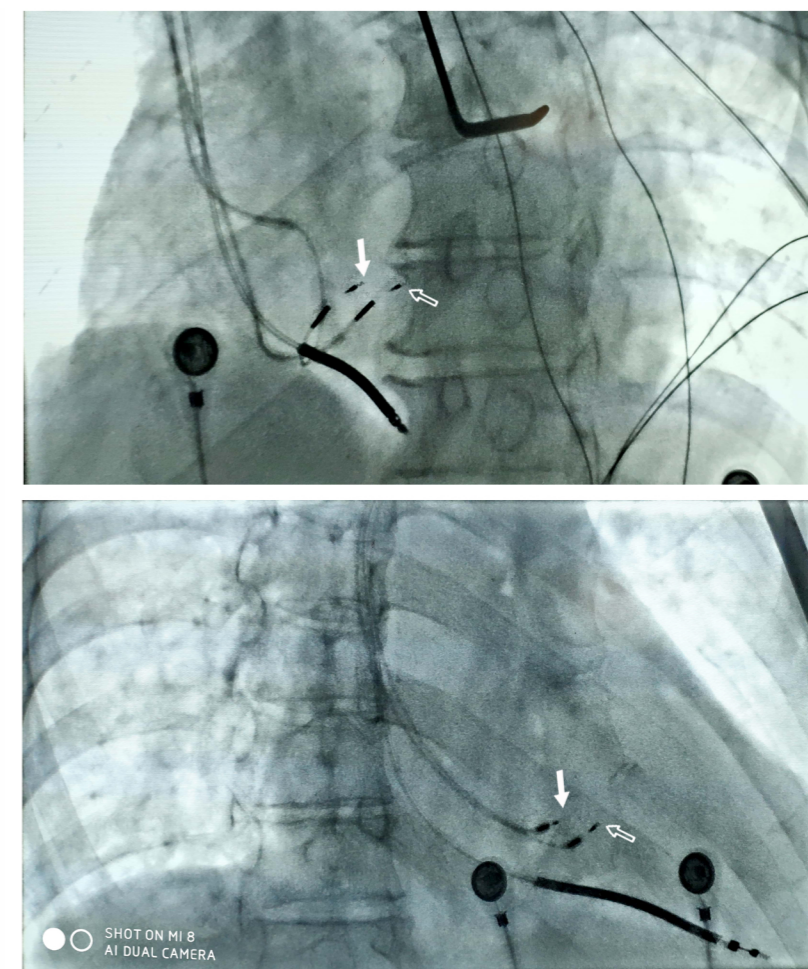

B

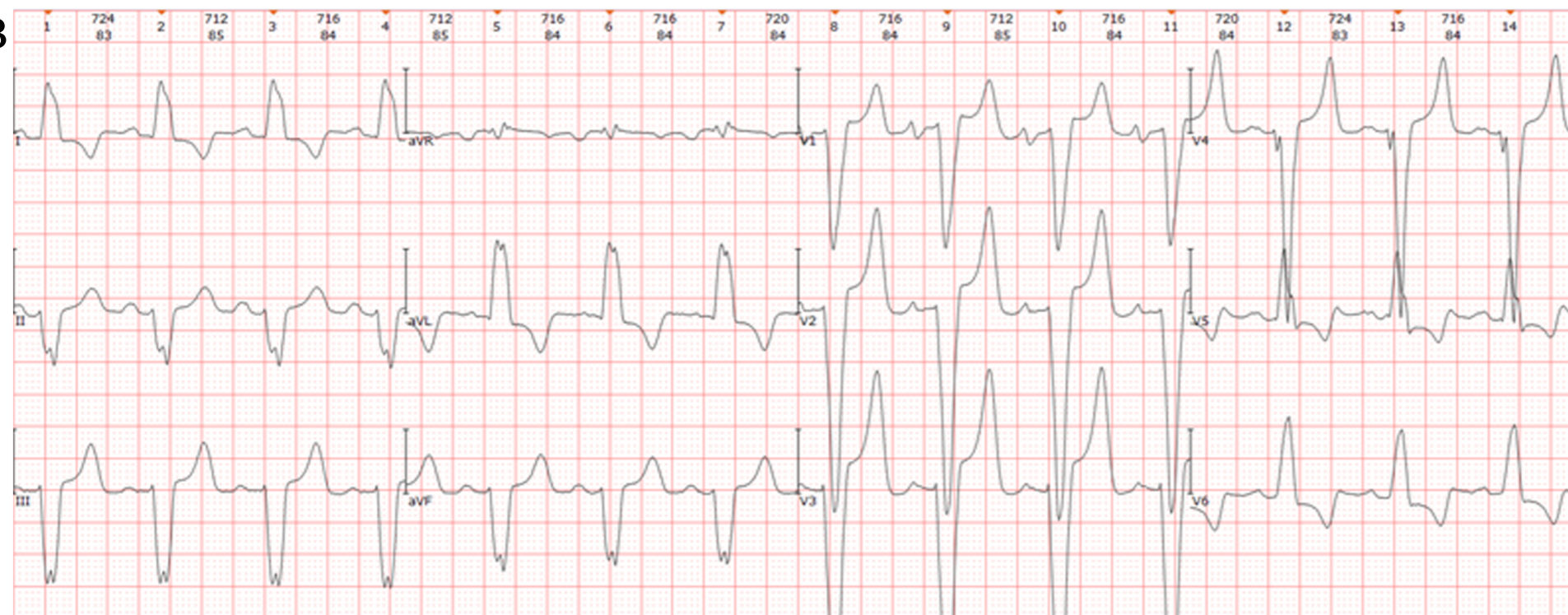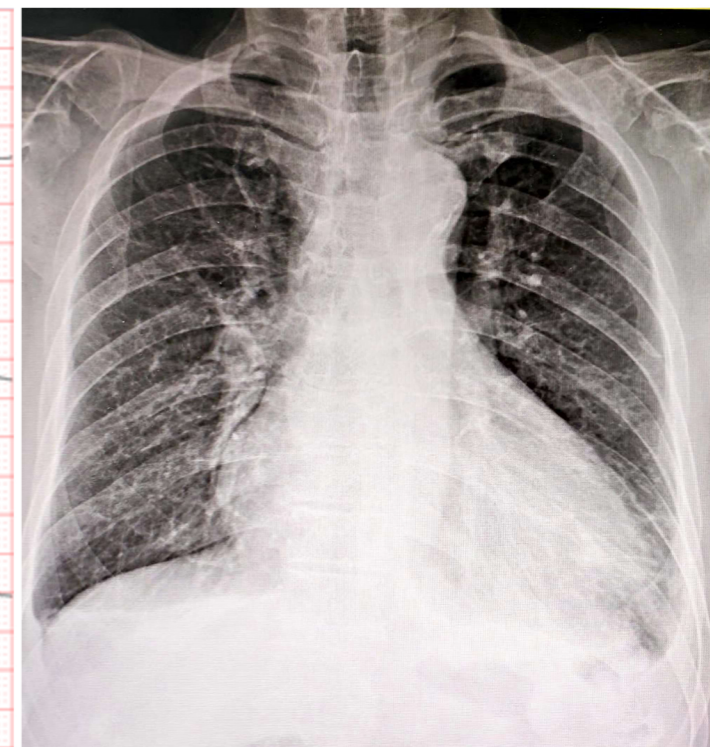

C

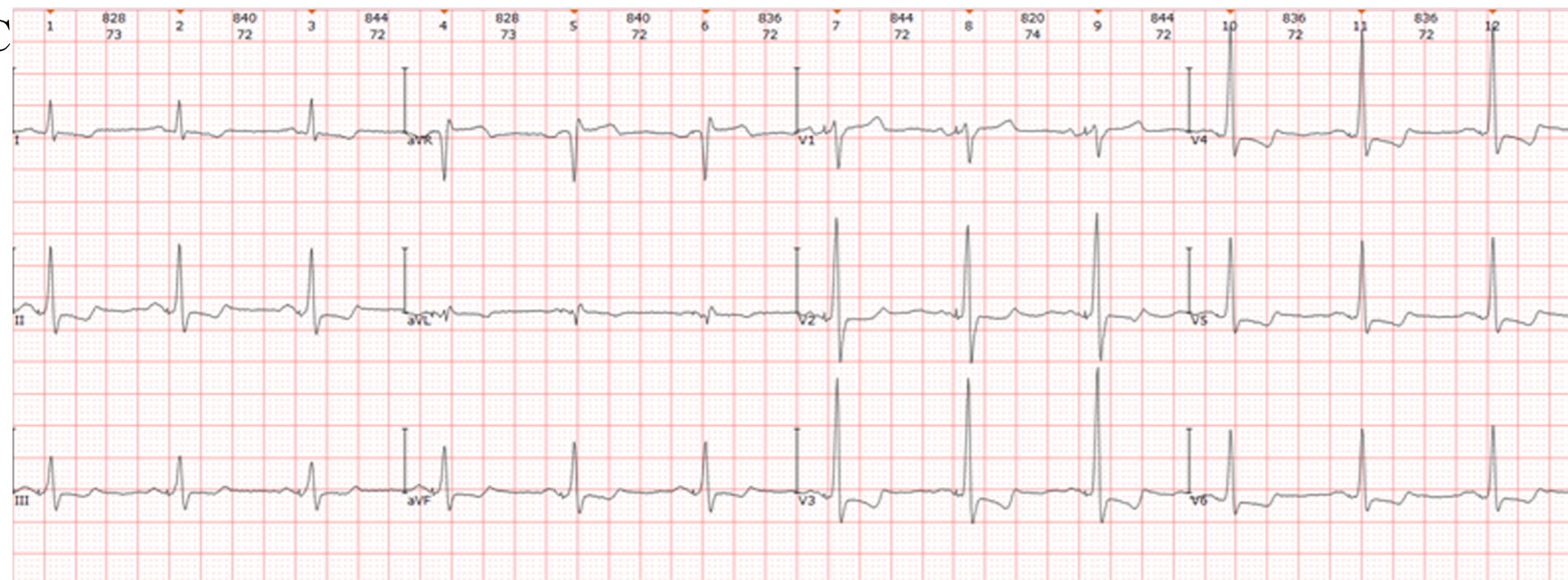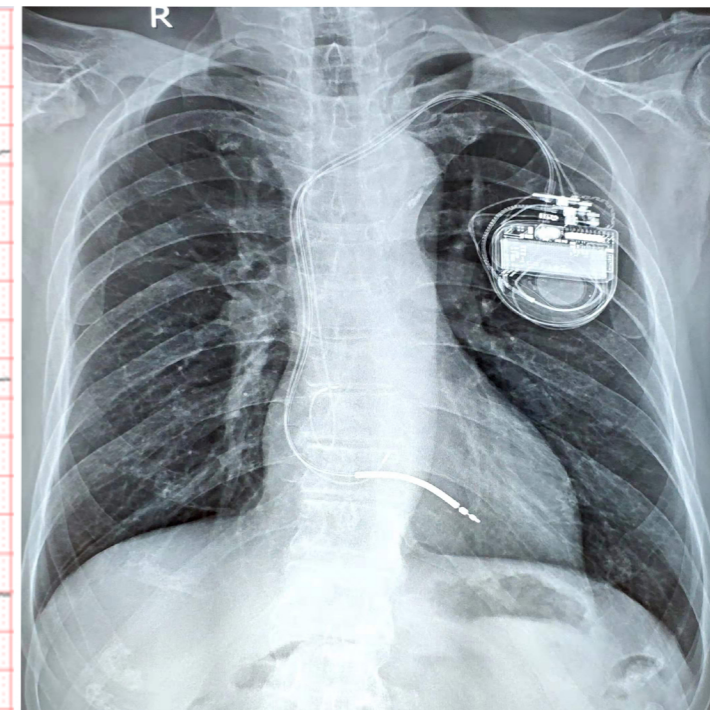

Supplement: Supplementary file 2 — Figure S1 LBBP‐CRT in a patient with NICM and strictly defined LBBB A 65‐year‐old male heart failure patient presented a strict LBBB morphology. (a) the QRSd was 165 ms at baseline. LBBB was corrected under temporary HBP with high output (10 V@0.4 ms) and the stim‐LVAT was narrowed to 90 ms. LBBB could be corrected under high and low output with consistency in stim‐LVAT of 87 ms. Selective‐LBBP morphology presented with an isoelectric interval between the pacing spike and the QRS onset under low output, the HBP lead (solid arrow) and LBBP lead (hollow arrow) was shown under fluoroscopy. (b) The ECG morphology met the criteria for strict LBBB proposed by Strauss et al. (c) QRS duration narrowed from 165 ms to 116 ms under LBBP with SAVD 100 ms and the CTR significantly decreased from 0.66 to 0.48 with LVEF improved from 32% to 69% at 3‐month follow‐up. NICM: non‐ischemic cardiomyopathy; LBBP: left bundle branch pacing; CTR: cardiothoracic ratio; LVEF: left ventricular ejection fraction; LVEDD: left ventricular end diastolic dimension, SAVD: sensed AV delay. [file CLC-43-1460-s002.pdf]

A

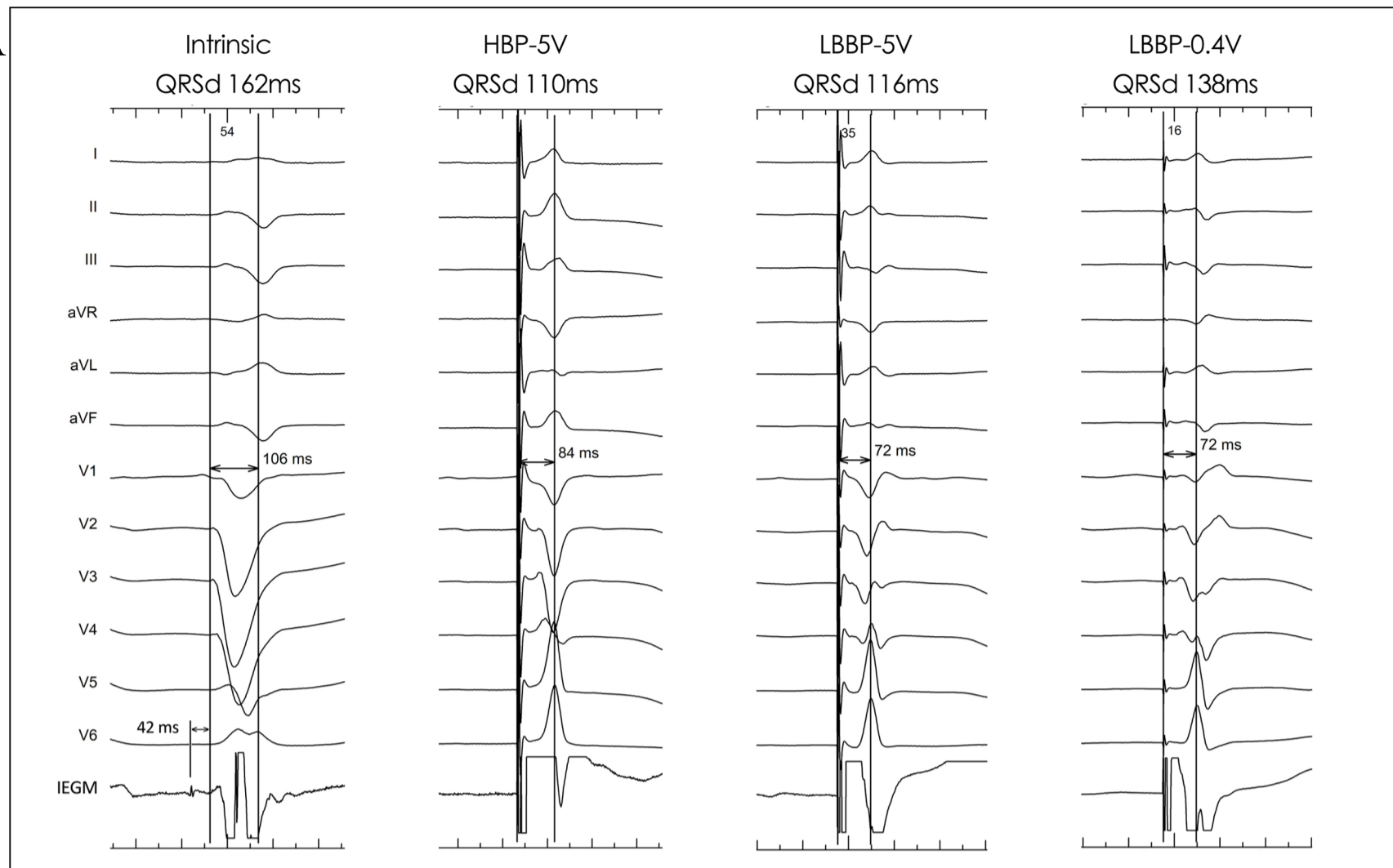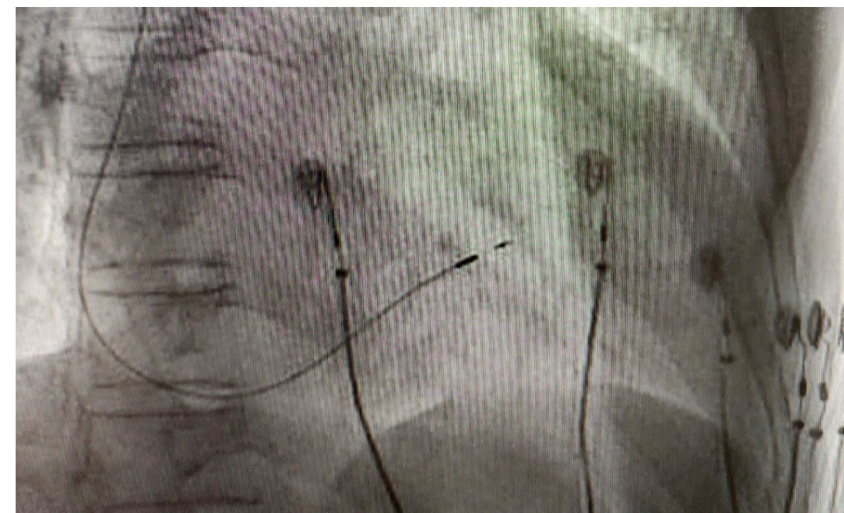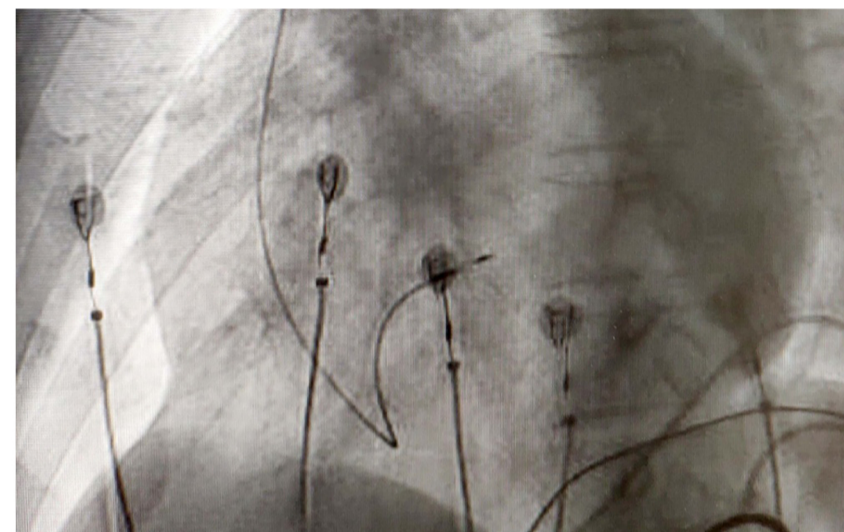

B

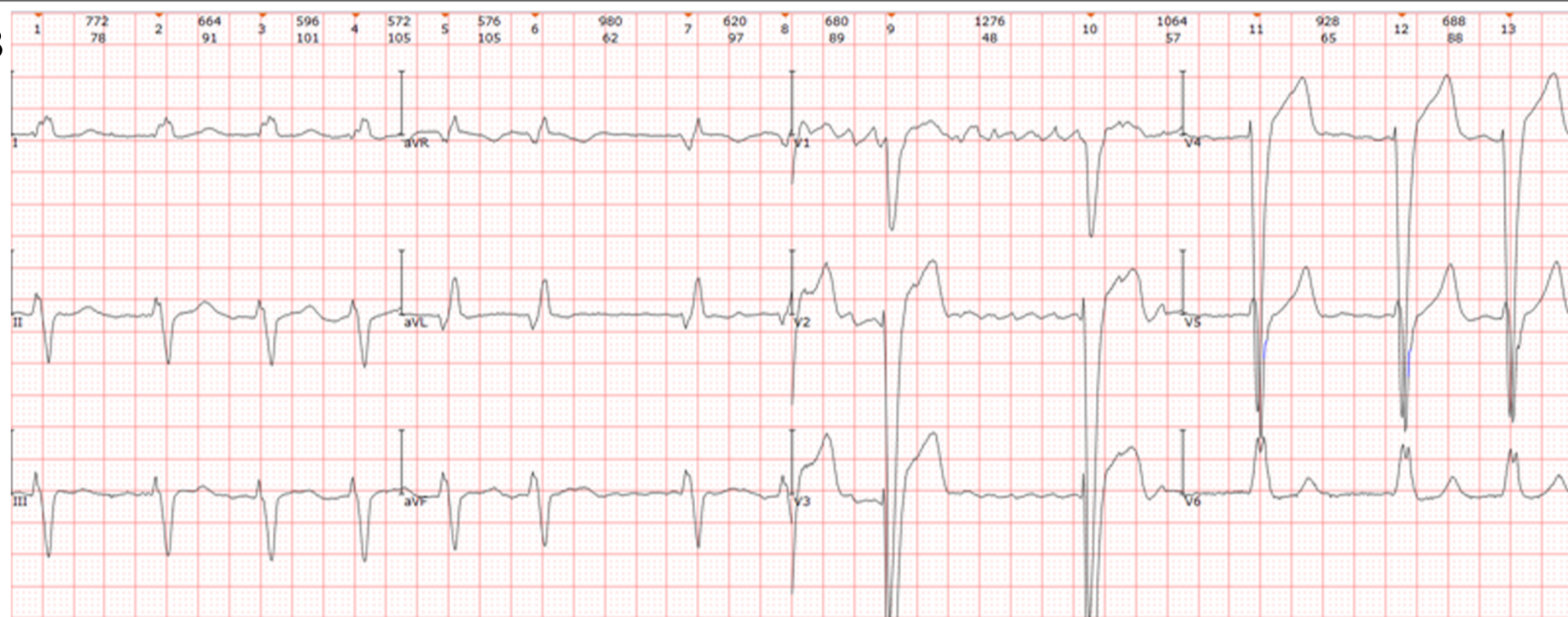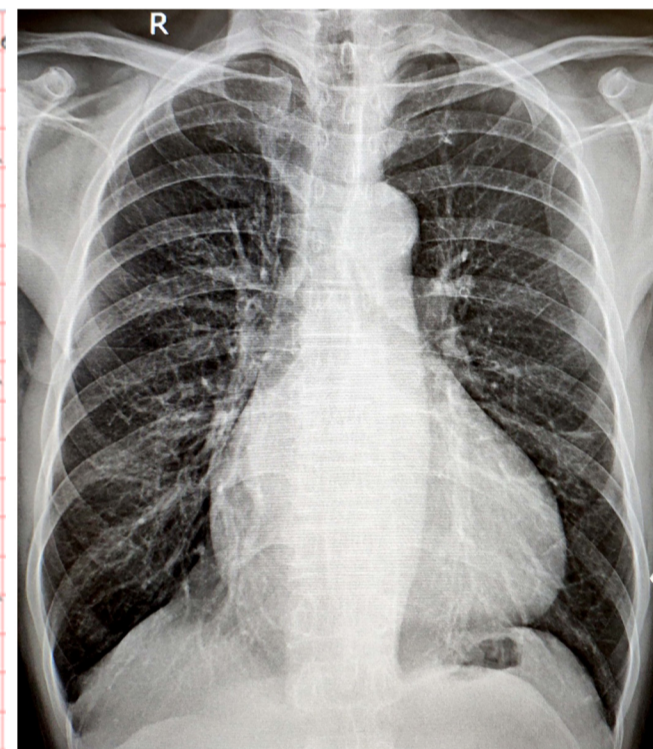

C

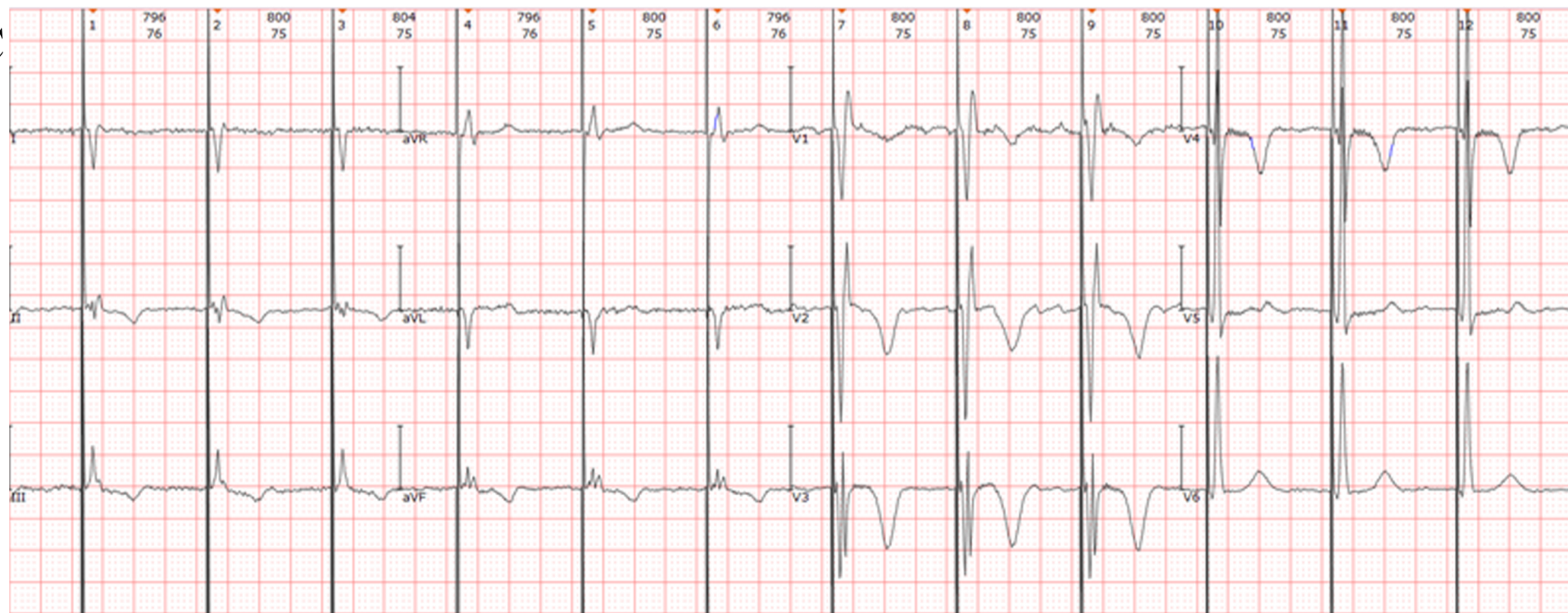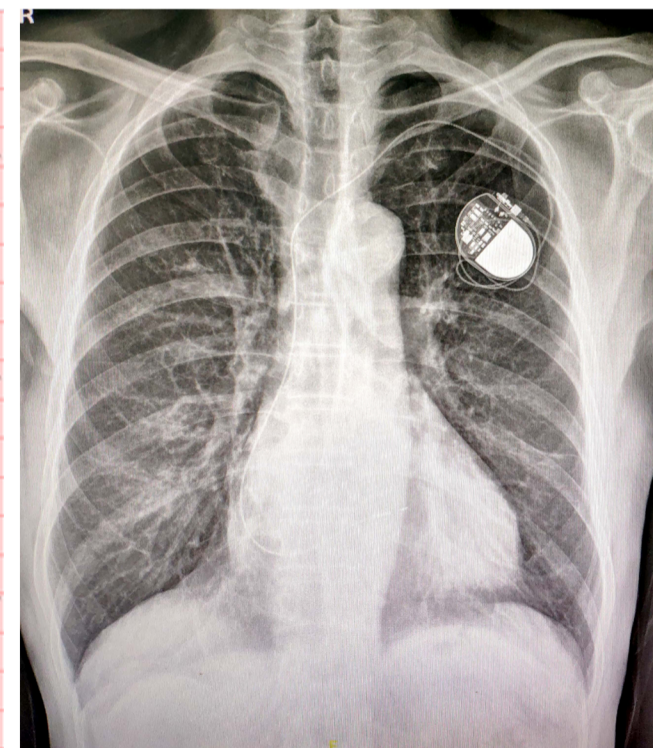

Supplement: Supplementary file 3 — Figure S2 LBBP‐CRT procedure in a patient with VVI implantation A 48‐year‐old male heart failure patient complicated with atrial fibrillation received LBBP‐CRT procedure with VVI implantation due to poor economic condition. (a) a strict LBBB morphology presented with QRSd of 162 ms. LBBB could be corrected by HBP under high output (5 V@0.4 ms) and stim‐LVAT was 84 ms. LBBB could be corrected by LBBP under high output (5 V@0.4 ms) and low output (0.4 V@0.4 ms) with consistency in stim‐LVAT of 72 ms. (b) The ECG showed atrial fibrillation and strict LBBB morphology (c) QRS duration was narrowed from 162 ms to 114 ms under LBBP and the CTR decreased from 0.59 to 0.49 with LVEF improving from 35% to 57% at 6‐month follow‐up. [file CLC-43-1460-s003.pdf]

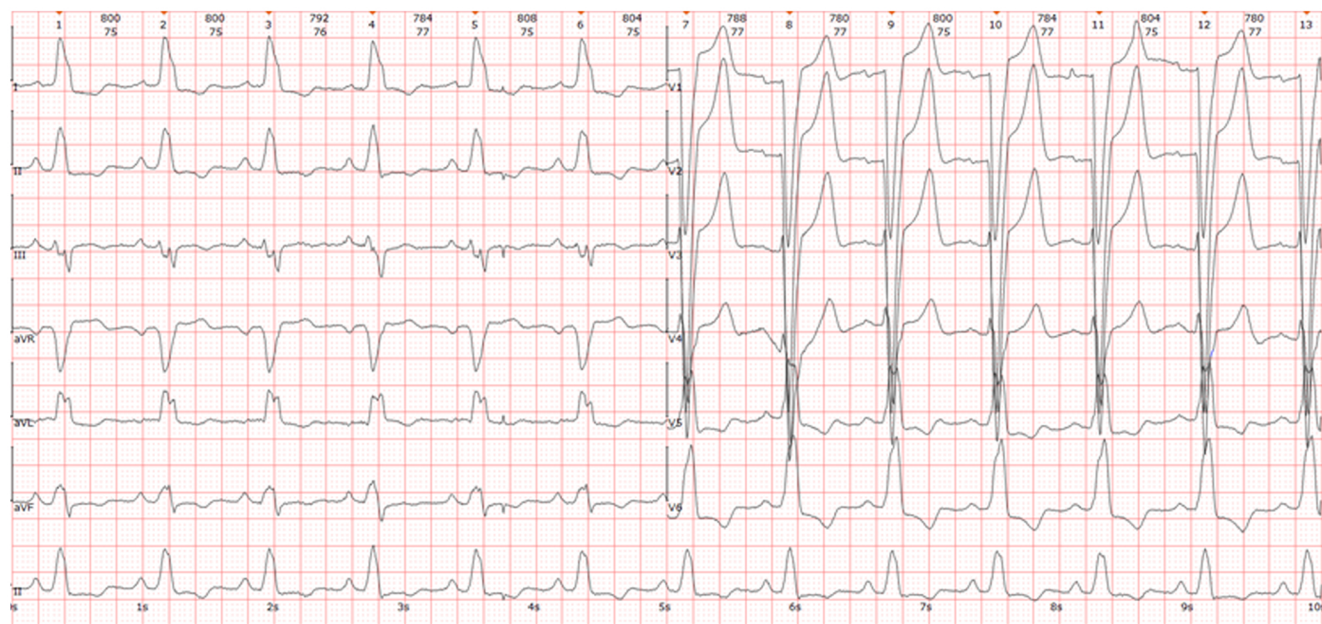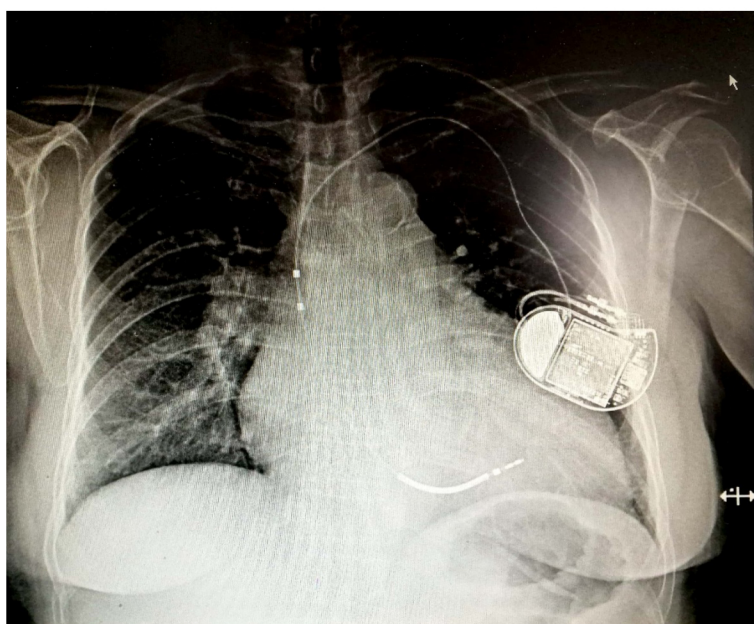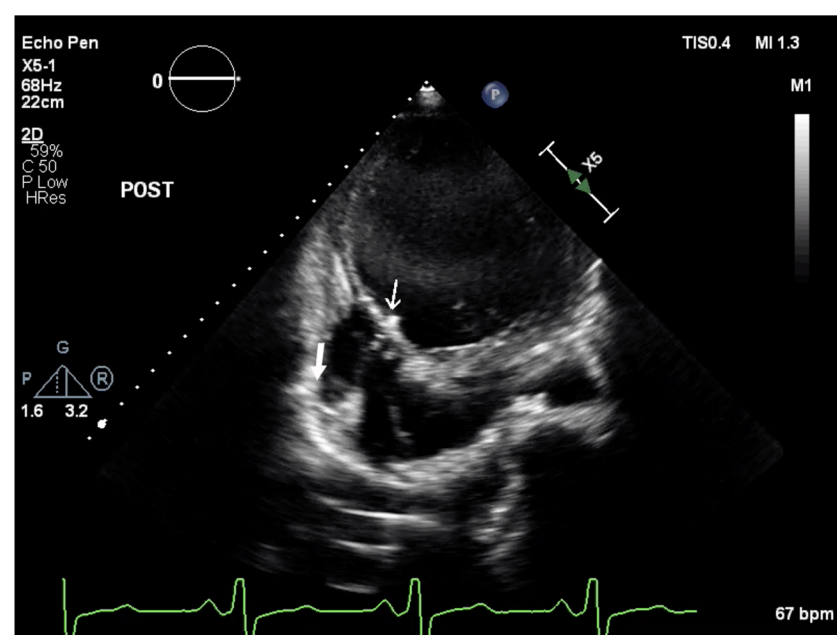

A

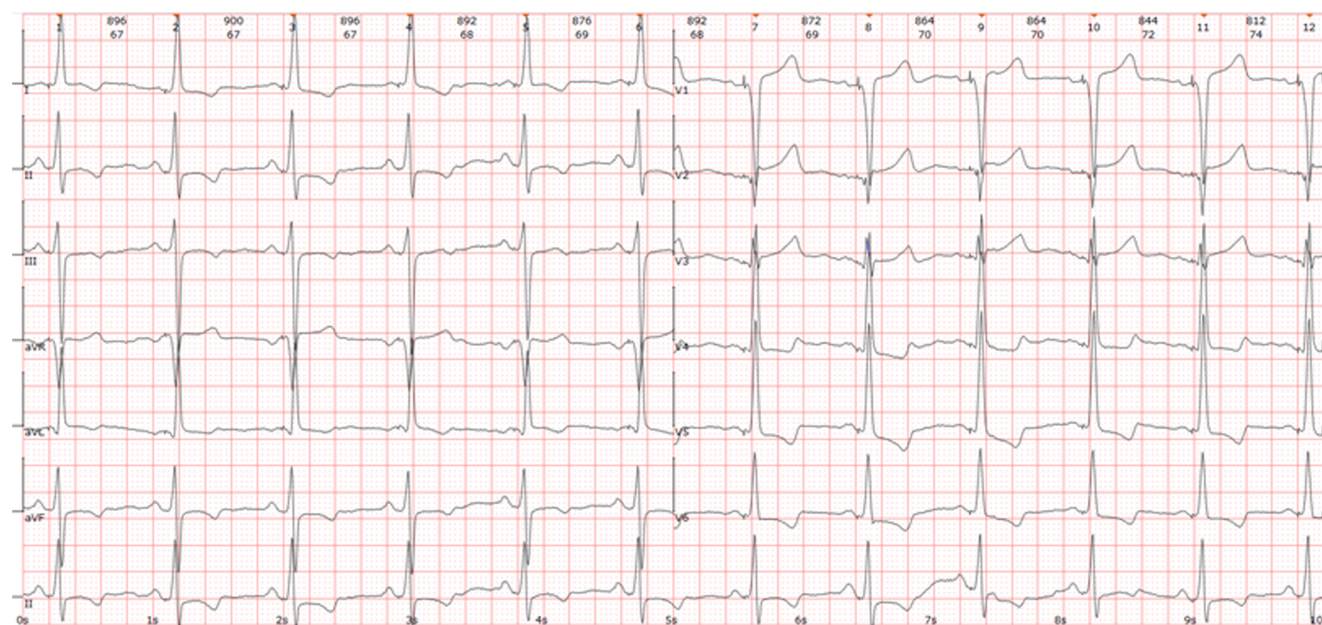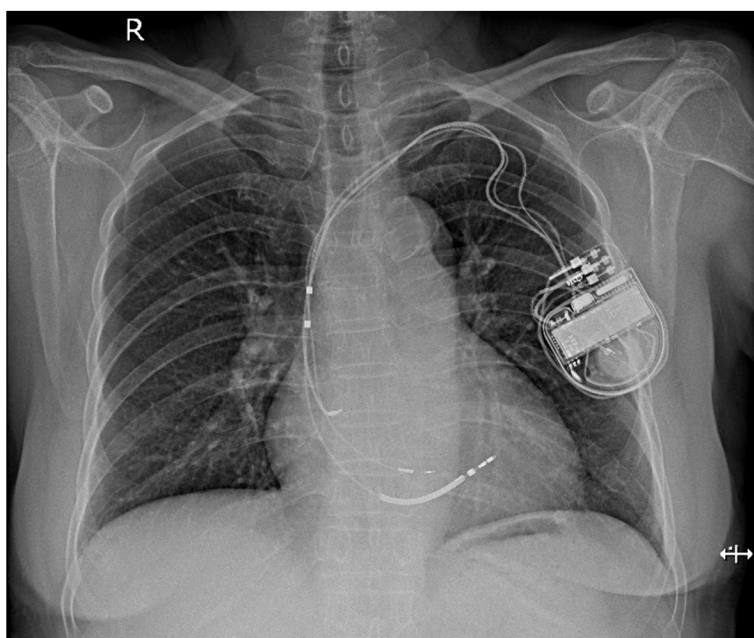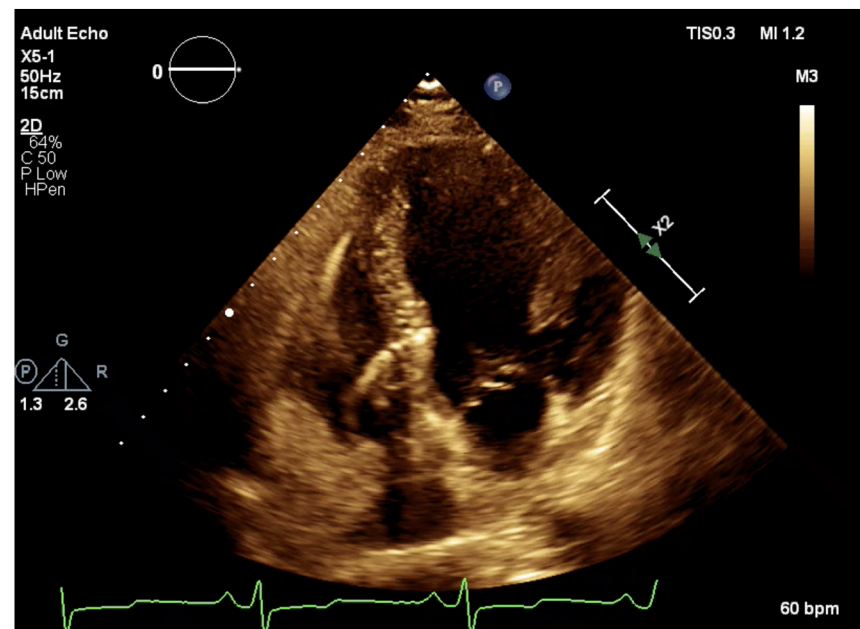

B

Supplement: Supplementary file 4 — Figure S3 Septal remodeling in a patient after LBBP‐CRT procedure A patient with NICM and LBBB demonstrated super response to LBBP. The QRSd was narrowed from 198 ms to 120 ms and the CTR decreased from 0.65 to 0.50.The phenomenon of “lead protruding” toward the LV cavity disappeared after remodeling of the ventricular septum during follow‐ups. The patient's IVSd increased from 7.8 mm to 10.2 mm. A: pre‐operation; B: post‐operation. LBBP: left bundle branch pacing; CTR: cardiothoracic ratio; LBBB: left bundle branch block; IVSd: interventricular septal diameter; NICM: non‐ischemic cardiomyopathy. QRSD: QRS duration. [file CLC-43-1460-s004.pdf]
